# Small molecule drug development for rare genodermatoses – evaluation of the current status in epidermolysis bullosa

**DOI:** 10.1186/s13023-020-01467-9

**Published:** 2020-10-19

**Authors:** Verena Wally, Manuela Reisenberger, Sophie Kitzmüller, Martin Laimer

**Affiliations:** 1 EB House Austria, Research Program for Molecular Therapy of Genodermatoses, Department of Dermatology and Allergology, University Hospital of the Paracelsus Medical University, 5020 Salzburg, Austria; 2 Department of Dermatology and Allergology, University Hospital of the Paracelsus Medical University Salzburg, Austria, 5020 Salzburg, Austria

**Keywords:** Epidermolysis bullosa, Genodermatosis, Small molecules, Orphan drugs, Clinical trial, Case study

## Abstract

**Background:**

Hereditary epidermolysis bullosa (EB) comprises a heterogeneous group of rare genodermatoses, which are caused by mutations in genes involved in the maintenance of the structural and functional integrity of dermo-epidermal adhesion in various stratified epithelia. In severe variants, generalized skin disease, extracutaneous manifestations and multi-organ involvement cause considerable morbidity and mortality. Causal and early treatment by re-expression of a respective mutated gene is the major long-term goal in therapy development. However, characterization and targeted modulation of pathogenic molecular cascades in EB also holds great promise as a symptom-relieving approach to ameliorate phenotype, complications and quality of life. Small molecules are chemical structures of less than 900 Da that can diffuse across cell membranes and interfere with target biomolecules, thus influencing their function at different levels. They constitute the vast majority of active components of all approved drugs.

**Methods:**

We performed PubMed and Google Scholar search for publications and screened FDA- and EMA-hosted clinical trial registries to identify studies using small molecule-based drugs for epidermolysis bullosa. Upon detailed analysis this resulted in the identification of a total of 84 studies.

**Results:**

We identified 52 publications and 32 registered trials that investigate small molecules for their safety and efficacy as treatment for different aspects of epidermolysis bullosa. Further, a total of 38 different small molecules clinically used in EB were found. Most frequent outcome measures concerned wound healing, reduction in blister numbers, as well as reduction of itch and pain, predominantly for EBS and RDEB.

**Conclusion:**

We provide a comprehensive summary of the current status of clinical small molecule development for EB and discuss prospects and limitations in orphan drug development for rare conditions like EB.

## Introduction

Recent advances in understanding pathomechanisms of different epidermolysis bullosa (EB) subtypes have facilitated the development of novel, targeted therapeutics. These approaches address debilitating symptoms of EB, like recurring or chronic wounding, pain, pruritus, failure to thrive and carcinogenesis. We herein summarize latest advances in repurposing or development of small molecule-based treatments targeting pathogenic traits in EB, while the use of biologics, as well as gene- and cell-therapeutic approaches will not be discussed.

### The heterogeneity of EB-symptoms and complications

The term EB comprises a group of rare, clinically and genetically heterogeneous genodermatoses characterized by moderate to excessive fragility of epithelial tissues and prototypic blistering following minimal trauma. EB is caused by mutations in genes encoding components important for the structural and functional integrity of the basement membrane zone in skin and mucous membranes [1, 2]. Genotypic heterogeneity as well as epigenetic, environmental and socio-economic factors contribute to the broad clinical spectrum and complex genotype-phenotype correlations [1].

The expression of distinct EB-associated genes in diverse epithelialized or mesenchymal organs explains extracutaneous involvement. The latter is particularly prominent in the severe subvariants, rendering EB a multi-system disease with significant morbidity and mortality due to relevant complications such as malnutrition, infections, organ failure and skin cancer [1].

Chronic tissue damage with induction and dysregulation of inflammatory pathways is a common pathogenic mechanism in EB as a mutation-based barrier disorder, although inter- and intra-individual variability of immune responses remains to be determined [3–7].

While in localized EB variants inflammatory aberrations mainly affect the micromilieu of lesional skin (leading to tissue remodeling [4–7]), a systemic impact was shown in severe subtypes such as RDEB, in which extensive cutaneous involvement is associated with a systemic inflammatory response and chronification of inflammatory cascades that contribute to the systemic morbidity of EB [8–12]. Against this background, the induction of inflammatory traits defines new therapeutic targets including skin barrier restoration, infection control/surveillance, immune response/modulation, anti-neoplastic interference and interference with epigenetic drivers of the disease. In this context, small molecule drugs provide clinical perspectives to mitigate the phenotype as well as complications.

### The potential of targeted small molecule drugs

Based on the increasing knowledge on EB subtype-specific pathomechanisms, first candidates have reached clinical development, and first marketing approvals are awaited in the near future. Drugs that tackle unspecific disease symptoms like pruritus and pain have been used in EB for a long time. However, the difference to currently developed drugs is their direct targeting of defined and known EB-subtype-related targets. This renders them more specific and potentially more efficient and safe [13, 14].

**Fig. 1:**
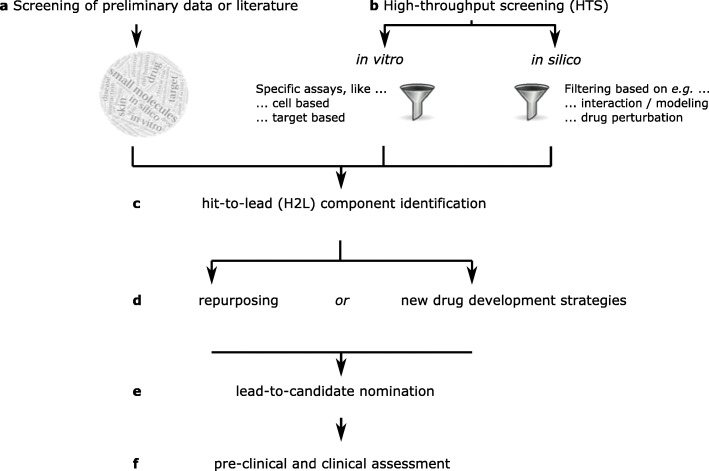
Simplified scheme of drug development options. **a** While screening of preliminary data or literature restricts number of hits (candidates that potentially interfere with a predefined target or pathway) to such already published or rationally identified, **b** high-throughput screening (HTS) can screen thousands of compounds simultaneously. The latter can be done in vitro using predefined assays with clear read-outs in combination with drug libraries, as well as in silico based on big data to identify drug-drug or drug-disease similarities, or using bioinformatic modeling. **c** Both approaches result in a number of hits, out of which lead candidates for further development are selected upon further confirmatory testing. **d** For lead components, further predefined testings are performed, which are amongst others dependent on whether a drug is already approved for other conditions or if the whole drug development process has to be performed. **e** Resulting candidates can then be taken forward to pre-clinical testings first, and if passing all exigencies, to clinical assessment (**f**)

Small molecules can interfere with a plethora of biomolecules, and are mostly designed to impair or alter target protein function. Approaches to identify candidates for drug repurposing are either rational and literature-based, or rely on the concomitant evaluation of hundreds of small molecules in high throughput screening (HTS) settings. For HTS, the design of a highly reliable, reproducible and biologically relevant assay with a representative and simple phenotype- or target-based read-out is a pre-requisite for successful HTS (Fig. 1a,c-f).

More recent developments have led to the integration of publicly available big data (e.g. transcriptomes) as an attractive source for drug development/repurposing and the advances of in silico 3D-modelling [13]. Used algorithms have reached a very high complexity and can integrate a huge amount of data, so that these platforms have the potential to translate into meaningful lead candidate nomination (Fig. 1b-f).

The classical pathway of further drug development comprises pre-clinical studies including e.g. toxicity and potency studies, formulation, pharmacokinetics. Clinical development comprises study phases I to III, with a final registration study. In the case of repurposing, single phases may be skipped under certain circumstances, which is an advantage that lines up with a reduced risk of failure due to already existing safety data, the subsequent reduced time of development, and potentially reduced development costs.

## Methods

From September 2019 to April 2020, we conducted a literature review using the PubMed and Google Scholar databases. Search terms included combinations of “epidermolysis bullosa”, “small molecules”, “clinical trial”, “case study”, and “drugs”. In addition, the clinical trial databases of the FDA [15] and EMA [16] were searched for trials for epidermolysis bullosa. Publications and trials investigating drugs in a clinical setting were included, those investigating biologics, cell- or gene-therapeutic approaches were excluded. In addition, non-English-language publications, review articles and pre-clinical studies were excluded. Following these inclusion and exclusion criteria, a total of 52 publications and 32 registered clinical trials were selected for evaluation in this review (Suppl. Figure 1).

## Results

Publications describing first attempts of drug repurposing for EB date back to the 80s, and have been used even long before ([17–54] Additional file 2). The following section gives an overview of the latest publications (2015-) on (randomized)-controlled trials ((R)CT) and currently registered active clinical studies (Additional file 3) in which small molecules are investigated for their potential utility in EB. Small molecule drugs that are currently being developed at a pre-clinical stage will not be discussed. Overall, publications on 28 different small molecules clinically used in EB were found, and 20 in registered clinical trials, with an overlap of 10 drugs.

### Summary of publications on recent (R)CT-trials and current clinical development status

Gentamicin is an aminoglycoside antibiotic, which has been applied in several genetic disorders such as cystic fibrosis [55] and hemophilia [56]. Aminoglycosides promote stop codon read-through by enabling the ribosome to pass the stop codon in the mRNA resulting in restored protein translation [57]. In a RCT trial, five RDEB patients with nonsense mutations in the *COL7A1* gene were treated with either topically applied or locally injected gentamicin, which induced anchoring fibril formation, improved wound closure and reduced blister numbers [58]. Further studies using topical formulations in JEB reported improved wound healing and reduction in blisters numbers [59, 60]. In a retrospective study including five JEB patients, the systemic application of gentamicin was assessed for the first time. Gentamicin was applied intravenously (*i.v.*) followed by intra-muscular (*i.m.*) injection, or *i.m.* only, leading to improved skin stability and quality-of-life (QoL) in 4/5 patients. However, in this study it did not postpone lethality [61]. Currently, two trials involving gentamicin treatment are running (NCT04140786, NCT03526159), aiming to increase laminin-332 and C7 expression in order to induce the generation of new hemidesmosomes and anchoring fibrils, respectively, monitored by immune- and electron microscopy of skin biopsies. Topical and intravenous applications are compared.

Betulin: Betulin-enriched triterpene extract is mainly isolated from birch bark and is already approved and used for partial thickness wounds. A semisolid gel formulation was used in a phase II, reference therapy-controlled trial, in which long-standing and acute RDEB-wounds were topically treated and analyzed for enhanced re-epithelialization [62]. Betulin mainly effects the inflammatory phase of the wound healing process, where it positively modulates mediators of inflammation, like COX-2 or IL-6 [63]. In addition, it is also thought to have anti-cancer effects [64, 65]. While it was shown that the formulation was well tolerated, only a slight, statistically not significant effect on wound healing was observed. However, results encouraged a larger clinical trial that is currently ongoing (NCT03453632), where betulin is tested as topical formulation for its efficacy and safety in EB. Results of verum and placebo groups will be compared by evaluating answers to the “Foot Health Status Questionnaire”.

Diacerein: For the sev-EBS, which is caused by dominantly inherited mutations within either *KRT5* or *KRT14*, it was shown that a pro-inflammatory signaling-cascade triggered by aggregation of mutated keratins is linked to skin fragility. In a positive feedback loop, IL-1ß is activated and released from effected keratinocytes and, besides paracrine signaling, enhances the expression of KRT14, resulting in a perpetuum mobile-like state [66]. Diacerein, a small molecule derived from the rhubarb root, can interfere with the IL-1ß mediated inflammatory pathway at several levels [67–73]. In a phase II/III RCT that was based on a previous pilot study [74], diacerein was shown to significantly reduce blister numbers in sev-EBS patients in a 4-week treatment episode. This effect further increased to the end of a 3-months follow-up [75]. After two investigator-driven trials, further studies using a 1% Diacerein-containing ointment were conducted. While one study was prematurely terminated, a further trial is ongoing which focuses on pharmacokinetics after long-term use (NCT03389308).

Serlopitant: In EB patients, itch is a major distressing symptom that worsens the disease phenotype due to scratching-induced wounding. The neurokinin-1 (NK1) receptor plays a major role in the pathogenesis of itch, and NK1-receptor antagonists, such as Serlopitant, are used to disrupt itch signaling [76]. In a RCT with 14 EB-patients, oral serlopitant led to itch reduction, although results were not statistically significant [77]. Currently, a larger clinical trial is ongoing with the aim to achieve a ≥ 3-points reduction in itch severity after 2 months of treatment (NCT03836001).

Epigallocatechin-3-gallate (EGCG): Matrix-metalloproteinase 7 (MMP7) is expressed at increased levels in the skin of RDEB patients, where it contributes to the degradation of C7 [78]. Therefore, the MMP7 inhibitor EGCG was used to treat 17 RDEB patients in an RCT-clinical trial with the aim to reduce blister numbers as well as itch, and to improve wound healing. Although a reduction in blister numbers was observed in 50% of patients compared to placebo, statistical analysis showed no significant difference [79]. Currently, no further trials are registered.

### Further small molecule drugs in currently ongoing clinical trials

Coenzyme Q10: In an open label trial (NCT02793960), a Coenzyme Q10 (ubidecarenone) cream is tested for its effect on wound healing upon application on at least one target wound and unaffected skin. The respective mode of action is to target mitochondria, e.g. via regulation of the cell metabolism during remodeling and expression of structural proteins, in order to strengthen the skin. Safety is controlled by blood tests, efficacy by changes in visual analog scale (VAS) pain scale and EBDASI questionnaires [80].

Botulinum toxin is frequently used for the treatment of wrinkles and hyperhidrosis, however, in the context of EB, Swartling and Holahan et al. reported on treating plantar blistering and pain in EBS patients. Intradermal injections led to a reduction of pain and reduced blister numbers, lasting for < 4 months [81, 82]. In a second case report, botulinum toxin was injected into the internal anal sphincter of an RDEB-patient over a 2-year period leading to relieved spasm and pain due to anal blistering, with a sustained effect lasting 4 years post treatment [83]. Following these studies, a currently recruiting intra-patient controlled trial (NCT3453632) evaluates the effect of botulinum toxin in a bigger patient cohort. The drug is injected into the sole of one foot, the other foot receives placebo. The effect in reduction of blister numbers is measured using an “Improvement Global Assessment” (IGA) score.

Pregabalin: This approved anticonvulsant is investigated to ameliorate pain and itch in RDEB patients. Via the inhibition of calcium currents, pregabalin reduces the release of neurotransmitters like glutamate, norepinephrine and substance-P in the central nervous system, and thus lowers neuronal excitability [84]. The systemic treatment is administered orally. The double-blinded cross over design allows the patients to serve as their own control. Primary outcome measures are patient reported pain scores (NCT03928093).

Sirolimus: Sirolimus is an mTOR inhibitor known for its anti-inflammatory effects [85]. In EB it is used to downregulate the translation of defective keratin proteins, therefore leading to an improvement of plantar skin lesions. Data are collected from a pedometer and lesion measurements with 3D-photography (NCT02960997).

Rigosertib: Squamous cell carcinomas (SCC), one of the most threatening co-pathologies in RDEB, is treated with Rigosertib, a polo-like kinase (PLK)-1 inhibitor leading to apoptosis in cancer cells [86]. Oral and *i.v.* applications are available. Tumor progression is determined via RECIST (response evaluation criteria in solid tumors) evaluation and biomarker analysis (NCT03786237).

Ropivacaine: Ropivacaine, a local anesthetic, reduces pain via impulse conduction blockage through inhibition of the sodium ion influx in nerve cells [87]. In this trial (NCT03730584) it is applied on painful wounds before dressing change. Patient or caregiver indicates pain levels during the procedure using respective analogue scale.

In summary, while most published clinical studies used oral administration (oral: 30/52; topical: 16/52, others: 8/52), in most currently registered trials drugs are applied topically (21/32, oral: 9/32). This may reflect the striving to develop drugs that can be administered by the patient himself and at the same time to avoid side effects upon oral administration.

When analyzing outcome measures we found that nearly all studies could be assigned to either of 5 groups: a) improvement of wound healing, b) reduction of blister numbers, c) reduction of itch and d) pain, and e) the prevention or treatment of SCC. Here, the most striking differences are evident in the proportion of the endpoints wound closure versus reduction of blister numbers (published: 21% / 56%; registered: 75% / 25%).

**Fig. 2:**
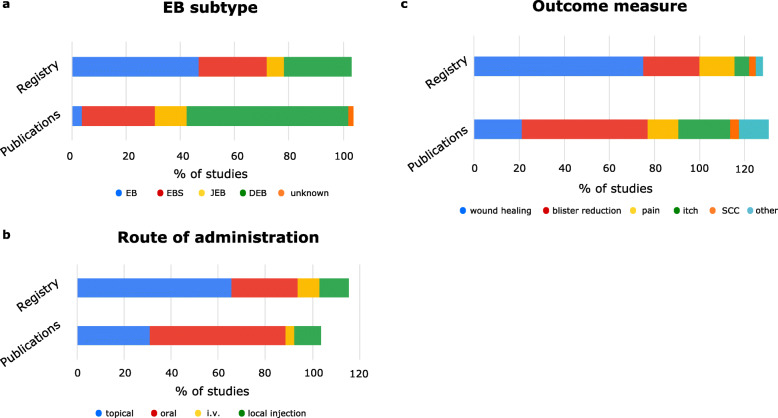
Summary of distribution of EB subtypes, outcome measures and routes of administration in reviewed trials. Percentage of respective parameters (**a**. EB subtype. **b**. outcome measure, **c**. route of administration) is given. A total of > 100% derives from the fact, that some studies have multiple specifications per criteria (e.g. outcome measure: wound healing and reduction of pain)

Finally, differences in EB subtypes included differs between publications and registered trials (registered: 15/32 open for all EB subtypes, whereas 49/52 published trials included only distinct subvariants), pointing towards the aim of industry to generate sufficient evidence for benefit as well as broad patient applicability of innovative therapeutics, whereas investigator-driven trials tend to focus on very specific and individual symptoms (Fig. 2, Table 1).

**Table 1:** Table giving the absolute numbers of studies investigating selected criteria (EB subtype, route of administration, outcome measure). As some studies list two possibilities for distinct criteria, respective sums might exceed the total of studies

	Publication (***n*** = 52)	Database (***n*** = 32)
**Outcome measure**		
**Wound healing**	11	24
**Blister reduction**	29	8
**Pain**	7	5
**Itch**	12	2
**SCC**	2	1
**Other**	7	1
**Route of administration**		
**Topical**	16	21
**Oral**	30	9
**i.v.**	2	3
**Local injection**	6	4
**Unknown**	2	0
**EB Subtype**		
**EB**	2	15
**EBS**	14	8
**JEB**	6	2
**DEB**	31	8
**unknown**	1	0

The positive outcome of several studies in reducing secondary disease manifestations will encourage the use of these drugs in the future. Some medications are already in advanced-phase trials and might enter the market within the next decade. The availability of symptom-relieving drugs that have been systematically tested for EB patients will likewise offer new treatment modalities, such as stand-alone treatments for milder subvariants or distinct complications, as well as combination regimes with other drugs/biologics or causal treatments approaches like gene-therapy. In this context, a consensus in the EB expert community on the complementarity of different therapeutic approaches to optimize treatment outcomes was recently reported, involving e.g. pain-relieving drugs such as opioids with non-pharmacological interventions [88, 89].

## Discussion

Currently, no general curative therapy is available for EB. In light of the morbidity and lethality of numerous EB-subvariants, causative therapeutic approaches, like gene-, protein-, and cell-based therapies, are urgently awaited. While treatments like cDNA replacement and cell-therapy have entered clinical development, the availability of such treatments for all patients with EB is not yet in reach due to limitations in terms of feasibility, efficacy, specificity, and safety [90–93]. Against this background, investigating new or otherwise used drugs for their effectiveness in EB gives promising perspectives for treatment approaches that positively impact symptoms, increase QoL and prevent/treat severe complications.

Generated evidence on efficacy of small molecules from published clinical trials is generally limited, reflecting mostly intrinsic and methodological constraints in small populations, including sample size requirements, recruitment failures, restricted replication and statistical power, or shortcomings in determining clinically meaningful endpoints and valid outcome measures. When analyzing publications on RCT-trials for EB (*n* = 11), less than 50% reached statistically significant efficacy, even though all but one trial report a positive effect of the treatment. This is most likely due to that fact that recruitment requirements were only met in a minority of trials, although sample size calculations were done for 5/11 trials. Difficulties in patient recruitment were mentioned by nearly all authors, even though substances were well tolerated with favorable safety profile, and without reported severe adverse events. Likewise, sample size issues and failures to reach statistical significance may underline the fact that almost 10% of pharma-driven trials had to be prematurely terminated.

As awareness towards rare diseases is raising, specific programs and adaptions of guidelines for respective drug developments are established to address deficits and promote accessibility to innovative therapeutics for EB patients. These include new methodological strategies [94], fast track designations, or the possibility to receive an orphan drug status by agencies like FDA or EMA. The latter gives the holder certain advantages, like the creation of financial incentives to develop those drugs. These incentives are being increasingly perceived and by now 7/38 here discussed compounds (+ 1 not registered or published) have been designated as orphan drugs. Two (diacerein, betulin) have additionally received a fast-track designation.

In terms of patient-centricity of clinical research as key measure to optimize trail recruiting and adherence, most selected endpoints of the analyzed published studies reflect treatment needs that are reported to be as much patient-relevant as currently unmet [95]. In particular, these include itch (12/52), pain (7/52), wound closure (11/52) and blister reduction (29/52). The numbers in registered clinical trials are diverging, where 24/32 focus on wound healing, and eight on reduction of blister numbers (Fig. 2a). This may reflect the fact that while wound healing is a generally well accepted endpoint, no guidelines for analyzing blister numbers exist. This, however, may be a hurdle for the development of drugs that address the pathophysiology of blisters, which is different to chronic wounds.

In general, perspectives for symptomatic immunomodulation in EB (including the evaluation of agents approved/tested for other immunologically mediated diseases like atopic dermatitis) are promising. However, it remains to be determined to which extent individual immune profiling is necessary to translate repurposing approaches into an efficient, safe, feasible, and tolerable therapeutic rational. Most probably, a multistep targeting approach and combinatory regimens will be essential for sustained efficacy in EB. In addition, approaches may further focus on subtypes for which the risk-benefit-ratio for current state of the art gene therapies are not yet beneficial (e.g. EBS), as well as on those associated with particularly high burden and numerous complications (e.g. DEB), which is reflected in the studies here analyzed. Likewise, the high proportion of topically and orally administered drugs (used in ~ 90% of all trials) reflects the effort to increase feasibility and tolerability for the vulnerable EB cohort.

## Conclusion

In summary, recent clinical trials are based on increasing understanding of pathomechanisms, resulting in more targeted approaches with clearly defined mechanisms of action of the investigational product. This renders the probability to conduct successful clinical trials more likely, and increases the chance for further clinical development until marketing approval. While new drug entities will still be coming up for diverse diseases and also for EB, drug repurposing holds great potential and is foreseen a promising, additional route to go.

## Supplementary information

**Additional file 1.** PRISMA 2009 Flow Diagram.

**Additional file 2: Table S1.** Clinical trials published for the treatment of EB using small molecules. This table is a summary of published small molecules in the therapy of Epidermolysis bullosa. Publications are ordered by EB subtype and type of trial (RCT, CT, case study), most recent publications on top of each section. The benefit for the patient was clustered into the groups reduction of blister numbers, itch reduction, pain reduction, improvement of wound healing, prevention and treatment of SCCs, and others. Others include QoL (*n* = 1), relieve anal sphincter spasm and fissuring (*n* = 1), lower stricture indices in esophageal stenosis (*n* = 1), plasma phospholipid and fatty acid profiles (*n* = 1), and reduction of inflammation (*n* = 1). na: not applicable. * Primary or clinically relevant endpoints are given for RCT trials. ^#^ Indicates drugs that are currently under clinical investigation in recruiting or running registered trials (Table S2)

**Additional file 3: Table S2.** Currently registered clinical trials for EB investigation small molecule-based drugs. The table combines trials registered in the following databases: www.clinicaltrials.gov [15] and www.clinicaltrialsregister.eu [16]. Trials are ordered according to current status, subtype and clinical trial phase. Status definitions were adopted from www.clinicaltrials.gov. Recruiting: The study is currently recruiting participants. Active, not recruiting: The study is ongoing, and participants are receiving an intervention or being examined, but potential participants are not currently being recruited. Terminated: The study has stopped early and will not start again. Participants are no longer being examined or treated. Completed: The study has ended normally, and participants are no longer being examined or treated. **Unknown:** A study whose last known status was recruiting; not yet recruiting; or active, not recruiting but that has passed its completion date, and the status has not been last verified within the past 2 years. Completed registered trials that have subsequently been published are only listed in Table S1

## Data Availability

Not applicable.

## Abbreviations

EB: Epidermolysis bullosa
EBS: Epidermolysis bullosa simples
EMA: European Medicines Agency
FDA: Food and Drug Administration
JEB: Junctional epidermolysis bullosa
KRT: Keratin
RCT: Randomized controlled trial
RDEB: Recessive-dystrophic epidermolysis bullosa
