# Supplementary material for: Small molecule drug development for rare genodermatoses – evaluation of the current status in epidermolysis bullosa
Source: Orphanet J Rare Dis. 2020 Oct 19;15:292. doi: 10.1186/s13023-020-01467-9 (PMC7574495; doi:10.1186/s13023-020-01467-9)
Supplement: Supplementary file 1 — Additional file 1. PRISMA 2009 Flow Diagram. [file 13023_2020_1467_MOESM1_ESM.doc]

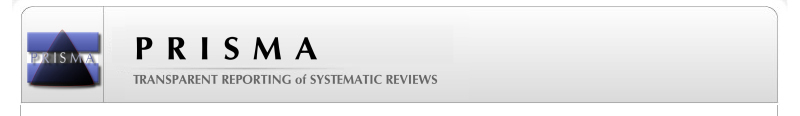
**PRISMA 2009 Flow Diagram**

**Screening**

**Included**

**Eligibility**

**Identification**

Records identified through database search
(“epidermolysis bullosa” “case study” n = 2266)

(“epidermolysis bullosa” “clinical trial” n = 94)

(“epidermolysis bullosa” “drug” n = 716)

(“epidermolysis bullosa” “small molecule” n = 7)

Additional records identified through other sources
(FDA and EMA clinical trial registries)
(n = 119)

Records (after duplicates removed)
(n = 97)

Studies included

(n = 84)

Records screened
(exclusion of publications and trials with biologics, cell or gene-therapeutic approaches, non-human) full text articles and registry entries only

(“epidermolysis bullosa” “case study” n = 2052)

(“epidermolysis bullosa” “clinical trial” n = 71)

(“epidermolysis bullosa” “drug” n = 672)

(“epidermolysis bullosa” “small molecule” n = 0)
(clinical trial registries: n = 32)

Records screened
clinical trials only

(“epidermolysis bullosa” “case study” n = 18)

(“epidermolysis bullosa” “clinical trial” n = 11)

(“epidermolysis bullosa” “drug” n = 12)

(“epidermolysis bullosa” “small molecule” n = 0)

(clinical trials registries

n = 32)

(total n = 73)

For all found substances, additional database search was performed to identify further publications in connectivity to epidermolysis bullosa

(n = 18)

Records excluded

(publications: n = 7)

(clinical trials: n = 0)
