# Supplementary material for: Small molecule drug development for rare genodermatoses – evaluation of the current status in epidermolysis bullosa
Source: Orphanet J Rare Dis. 2020 Oct 19;15:292. doi: 10.1186/s13023-020-01467-9 (PMC7574495; doi:10.1186/s13023-020-01467-9)
Supplement: Supplementary file 2 — Additional file 2: Table S1. Clinical trials published for the treatment of EB using small molecules. This table is a summary of published small molecules in the therapy of Epidermolysis bullosa. Publications are ordered by EB subtype and type of trial (RCT, CT, case study), most recent publications on top of each section. The benefit for the patient was clustered into the groups reduction of blister numbers, itch reduction, pain reduction, improvement of wound healing, prevention and treatment of SCCs, and others. Others include QoL (n = 1), relieve anal sphincter spasm and fissuring (n = 1), lower stricture indices in esophageal stenosis (n = 1), plasma phospholipid and fatty acid profiles (n = 1), and reduction of inflammation (n = 1). na: not applicable. * Primary or clinically relevant endpoints are given for RCT trials. # Indicates drugs that are currently under clinical investigation in recruiting or running registered trials (Table S2) [file 13023_2020_1467_MOESM2_ESM.docx]

| .Table 1 | **improvement of wound healing** | **reduction of blister numbers** | **itch reduction** | **pain reduction** | **prev. or treatment of RDEB-SCC** | **others** |  | | | | |
| --- | --- | --- | --- | --- | --- | --- | --- | --- | --- | --- | --- |
| **(Main) Active component** | **Benefit for patient or clinical endpoints*** | | | | | | **Application** | **Type** | **Controlled** | **Participants** | **Reference** |
| **EB** | | | | | | | | | | | |
| Serlopitant^#^ |  |  |  |  |  |  | oral | RCT | placebo | 14 | Chiou et al, 2019 ^39^ |
| Cannabinoid (JEB, RDEB) |  |  |  |  |  |  | oral, topical | case study | na | 3 | Schrader et al, 2019 ^59^ |
| Cannabinoid (EBS, RDEB) |  |  |  |  |  |  | topical | case study | na | 3 | Chelliah et al, 2018 ^62^ |
| Budesonide |  |  |  |  |  |  | topical | open-label trial | na | 6 | Zanini et al, 2014 ^68^ |
| **EBS** | | | | | | | | | | | |
| Diacerein^#^ |  |  |  |  |  |  | topical | RCT | placebo | 17 | Wally et al., 2018 ^37^ |
| Aluminium chloride hexahydrate |  |  |  |  |  |  | topical | RCT | placebo | 23 | Younger et al, 2007 ^74^ |
| Tetracyclin |  |  |  |  |  |  | oral | RCT | placebo | 12 | Weiner et al, 2004 ^78^ |
| Oxytetracycline |  |  |  |  |  |  | oral | RCT | placebo | 21 | Hansen et al, 1996 ^84^ |
| Bufexamac |  |  |  |  |  |  | topical | RCT | placebo | 10 | Fine et al, 1988 ^90^ |
| Diacerein^#^ |  |  |  |  |  |  | topical | open label followed by RCT | placebo | 5 | Wally et al, 2013 ^36^ |
| Erythromycin |  |  |  |  |  |  | oral | open-label trial | na | 6 | Chiaverini et al, 2015 ^64^ |
| Cyproheptadine |  |  |  |  |  |  | oral | controlled trial | no treatment | 13 | Neufeld-Kaiser et al, 1997 ^83^ |
| Onabotulinum toxin A^#^ |  |  |  |  |  |  | local injection | case study | na | 1 | Holahan et al, 2016 ^44^ |
| Abobotulinum toxin A^#^ |  |  |  |  |  |  | local injection | case study | na | 6 | Swartling et al, 2010 ^43^ |
| Abobotulinum toxin A^#^ |  |  |  |  |  |  | local injection | case study | na | 1 | Abitbol et al, 2009 ^73^ |
| Tetracycline |  |  |  |  |  |  | oral | case study | na | 2 | Retief et al, 1999 ^82^ |
| Isotretinoin |  |  |  |  |  |  | oral | case study | na | 1 | Andreano et al, 1988 ^89^ |
| **RDEB** | | | | | | | | | | | |
| Gentamicin^#^ |  |  |  |  |  |  | topical / local injection | RCT | placebo | 5 | Woodley et al, 2017 ^20^ |
| Epigallocatechin-3-gallate |  |  |  |  |  |  | oral | RCT | placebo | 17 | Chiaverini et al, 2016 ^41^ |
| Trimethoprim |  |  |  |  |  |  | oral | RCT | placebo | 10 | Lara-Corrales et al, 2012 ^71^ |
| Phenytoin |  |  |  |  |  |  | oral | RCT | placebo | 36 | Caldwell-Brown et al, 1992 ^87^ |
| Celecoxib |  |  |  |  |  |  | oral | case study | na | 1 | Reimer et al, 2020 ^58^ |
| Botulinum toxin A^#^ |  |  |  |  |  |  | local injection | case study | na | 1 | Chaptini et al, 2015 ^45^ |
| Budenoside |  |  |  |  |  |  | topical | case study | na | 2 | Dohil et al, 2011 ^72^ |
| Isotretinoin |  |  |  |  |  |  | oral | open-label trial | na | 20 | Fine et al, 2004 ^77^ |
| Phenytoin |  |  |  |  |  |  | oral | retrospective | no treatment | 13 | Cunnane et al, 1987 ^91^ |
| Etretinate |  |  |  |  |  |  | oral | case study | na | 1 | Fritsch et al, 1983 ^93^ |
| Phenytoin |  |  |  |  |  |  | oral | trial | no treatment | 17 | Bauer et al, 1980 ^95^ |
| **DDEB** | | | | | | | | | | | |
| Naltrexone |  |  |  |  |  |  | oral | case study | na | 3 | Pallesen et al, 2019 ^60^ |
| Calcipotriol |  |  |  |  |  |  | topical | case study | na | 1 | Guttmann-Gruber et al, 2018 ^10^ |
| Minocyclin |  |  |  |  |  |  | oral | case study | na | 1 | Leung et al, 2015 ^65^ |
| Cyclosporin |  |  |  |  |  |  | oral | case study | na | 1 | Calikoglu et al, 2002 ^79^ |
| Cyclosporin |  |  |  |  |  |  | oral | case study | na | 1 | Takahashi et al, 1996 ^85^ |
| Mizoribine |  |  |  |  |  |  | oral | case study | na | 1 | Takahashi et al, 1996 ^85^ |
| **DEB** | | | | | | | | | | | |
| Mycophenolate motefil |  |  |  |  |  |  | oral | RCT | reference therapy | 35 | El-Darouti et al, 2013 ^69^ |
| Betulin^#^ |  |  |  |  |  |  | topical | open-label, controlled trial | reference therapy | 10 | Schwieger-Briel et al, 2017 ^24^ |
| Sucralfate |  |  |  |  |  |  | topical | case study | na | 1 | Yasar et al, 2018 ^63^ |
| Thalidomide |  |  |  |  |  |  | oral | case study | na | 1 | Rani et al, 2018 ^61^ |
| Ketamine / amitriptyline |  |  |  |  |  |  | topical | case study | na | 1 | Mangold et al, 2014 ^66^ |
| Thalidomide |  |  |  |  |  |  | oral | case study | na | 2 | Ranugha et al, 2014 ^67^ |
| Thalidomide |  |  |  |  |  |  | oral | case study | na | 1 | Ozanic Bulic et al, 2005 ^75^ |
| Tacrolimus |  |  |  |  |  |  | topical | case study | na | 1 | Banky et al, 2004 ^76^ |
| Phenytoin |  |  |  |  |  |  | oral | case study | na | 4 | Sasidharan et al., 2002 ^80^ |
| Sucralfate |  |  |  |  |  |  | oral | case study | na | 5 | Marini et al, 2001 ^81^ |
| Tacrolimus |  |  |  |  |  |  | oral | case study | na | 1 | Carroll et al, 1994 ^86^ |
| Minocyclin |  |  |  |  |  |  | oral | case study | na | 2 | White et al, 1989 ^88^ |
| **JEB** | | | | | | | | | | | |
| Gentamicin^#^ |  |  |  |  |  |  | topical | open-label trial | no treatment | 3 | Kwong et al, 2020 ^22^ |
| Gentamicin^#^ |  |  |  |  |  |  | topical | case study | placebo | 1 | Li et al, 2020 ^21^ |
| Gentamicin^#^ |  |  |  |  |  |  | *i.v*. / local injection | retrospective | na | 5 | Hammersen et al, 2019 ^23^ |
| Phenytoin |  |  |  |  |  |  | unknown | case study | na | 1 | Rogers et al, 1983 ^92^ |
| Phenytoin |  |  |  |  |  |  | unknown | case study | na | 2 | Bergfeld et al, 1982 ^94^ |
| **unknown** | | | | | | | | | | | |
| Phenytoin |  |  |  |  |  |  | *i.v.* injection followed by oral therapy | case study | na | 1 | Oztekin et al, 2013 ^70^ |
