# Supplementary material for: Small molecule drug development for rare genodermatoses – evaluation of the current status in epidermolysis bullosa
Source: Orphanet J Rare Dis. 2020 Oct 19;15:292. doi: 10.1186/s13023-020-01467-9 (PMC7574495; doi:10.1186/s13023-020-01467-9)
Supplement: Supplementary file 3 — Additional file 3: Table S2. Currently registered clinical trials for EB investigation small molecule-based drugs. The table combines trials registered in the following databases: www.clinicaltrials.gov [15] and www.clinicaltrialsregister.eu [16]. Trials are ordered according to current status, subtype and clinical trial phase. Status definitions were adopted from www.clinicaltrials.gov. Recruiting: The study is currently recruiting participants. Active, not recruiting: The study is ongoing, and participants are receiving an intervention or being examined, but potential participants are not currently being recruited. Terminated: The study has stopped early and will not start again. Participants are no longer being examined or treated. Completed: The study has ended normally, and participants are no longer being examined or treated. Unknown: A study whose last known status was recruiting; not yet recruiting; or active, not recruiting but that has passed its completion date, and the status has not been last verified within the past 2 years. Completed registered trials that have subsequently been published are only listed in Table S1 [file 13023_2020_1467_MOESM3_ESM.docx]

| Table 2. | **improvement of**  **wound healing** | | **reduction of blister**  **numbers** | | **itch reduction** | | **pain reduction** | | **prev. or treatment**  **of RDEB-SCC** | | **others** | |  | | | | | |
| --- | --- | --- | --- | --- | --- | --- | --- | --- | --- | --- | --- | --- | --- | --- | --- | --- | --- | --- |
| **(Main) Active component** | | **Benefit for patient** | | | | | | | | | | | **EB subtypes** | **Application** | **Phase** | **Control** | **Participants**  **(planned or actual)** | **Registration no.** |
| **Recruiting** | | | | | | | | | | | | | | | | | | |
| Serlopitant | |  | |  | |  | |  | |  |  | EB | | oral | II | placebo | 40 | NCT03836001 |
| Betulin | |  | |  | |  | |  | |  |  | EB | | topical | III | placebo | 250 | NCT03068780 |
| Ropivacaine | |  | |  | |  | |  | |  |  | EB | | topical | na | N | 10 | NCT03730584 |
| Botulinum toxin | |  | |  | |  | |  | |  |  | EBS | | local injection | II / III | placebo | 25 | NCT03453632 |
| Pregabalin | |  | |  | |  | |  | |  |  | RDEB | | oral | III | placebo | 15 | NCT03928093 |
| Gentamicin | |  | |  | |  | |  | |  |  | RDEB | | topical & *i.v.* injection | I / II | N | 9 | NCT03526159 |
| Losartan | |  | |  | |  | |  | |  |  | RDEB | | oral | I / II | N | 30 | 2015-003670-32 |
| Rigosertib | |  | |  | |  | |  | |  |  | DEB | | oral & *i.v.* injection | II | N | 12 | NCT03786237 |
| Gentamicin | |  | |  | |  | |  | |  |  | JEB | | topical & *i.v.* injection | I / II | N | 6 | NCT04140786 |
| **Active, not recruiting** | | | | | | | | | | | | | | | | | | |
| Ubidecarenone | |  | |  | |  | |  | |  |  | EB | | topical | I | N | 11 | NCT02793960 |
| Diacerein | |  | |  | |  | |  | |  |  | EBS | | topical | II | N | 80 | NCT03389308 |
| Sirolimus | |  | |  | |  | |  | |  |  | EBS | | topical | II | placebo | 8 | NCT02960997 |
| **Completed** | | | | | | | | | | | | | | | | | | |
| Opioid | |  | |  | |  | |  | |  |  | EB | | topical | IV | placebo | - | NCT00231517 |
| Allantoin | |  | |  | |  | |  | |  |  | EB | | topical | III | placebo | 169 | NCT02384460 |
| Serlopitant | |  | |  | |  | |  | |  |  | EB | | oral | II | placebo | 14 | NCT02654483 |
| Diacerein | |  | |  | |  | |  | |  |  | EB | | topical | II | placebo | 9 | NCT03468322 |
| Allantoin | |  | |  | |  | |  | |  |  | EB | | topical | II | placebo | 48 | NCT02014376 |
| Allantoin | |  | |  | |  | |  | |  |  | EB | | topical | II | N | 8 | NCT00825565 |
| Diacerein | |  | |  | |  | |  | |  |  | EB | | topical | I | N | 20 | NCT03472287 |
| Sulforaphane | |  | |  | |  | |  | |  |  | EBS | | topical | I | placebo | 5 | NCT02592954 |
| Trimethoprim | |  | |  | |  | |  | |  |  | RDEB, JEB | | oral | II | placebo | 10 | NCT00380640 |
| Gentamicin | |  | |  | |  | |  | |  |  | RDEB | | topical & local injection | I / II | N | 6 | NCT03012191 |
| Gentamicin | |  | |  | |  | |  | |  |  | RDEB | | topical & local injection | I / II | placebo | 6 | NCT02698735 |
| Polyphenon E | |  | |  | |  | |  | |  |  | DEB | | oral | II | placebo | 18 | NCT00951964 |
| **Terminated** | | | | | | | | | | | | | | | | | | |
| Allantoin | |  | |  | |  | |  | |  |  | EB | | topical | III | N | 152 | NCT02670330 |
| Allantoin | |  | |  | |  | |  | |  |  | EB | | topical | II | N | 42 | NCT02090283 |
| Diacerein | |  | |  | |  | |  | |  |  | EBS | | topical | II | placebo | 80 | NCT03154333 |
| **Unknown** | | | | | | | | | | | | | | | | | | |
| Tetracyclin | |  | |  | |  | |  | |  |  | EB | | oral | IV | placebo | 20 | NCT00336154 |
| Erythromycin | |  | |  | |  | |  | |  |  | EB | | oral | II | N | 8 | NCT01340235 |
| Botulinum toxin A | |  | |  | |  | |  | |  |  | EBS | | local injection | II | placebo | 40 | NCT00936533 |
| Diacerein | |  | |  | |  | |  | |  |  | EBS | | topical | II | placebo | 50 | NCT02470689 |
| Sirolimus | |  | |  | |  | |  | |  |  | EBS | | topical | II | placebo | 8 | NCT03016715 |
